# Decreasing miRNA sequencing bias using a single adapter and circularization approach

**DOI:** 10.1186/s13059-018-1488-z

**Published:** 2018-09-03

**Authors:** Sergio Barberán-Soler, Jenny M. Vo, Ryan E. Hogans, Anne Dallas, Brian H. Johnston, Sergei A. Kazakov

**Affiliations:** SomaGenics, Inc., Santa Cruz, California, USA

**Keywords:** miRNA-seq, Sequencing bias, Small RNA sequencing, Small RNA library preparation

## Abstract

The ability to accurately quantify all the microRNAs (miRNAs) in a sample is important for understanding miRNA biology and for development of new biomarkers and therapeutic targets. We develop a new method for preparing miRNA sequencing libraries, RealSeq®-AC, that involves ligating the miRNAs with a single adapter and circularizing the ligation products. When compared to other methods, RealSeq®-AC provides greatly reduced miRNA sequencing bias and allows the identification of the largest variety of miRNAs in biological samples. This reduced bias also allows robust quantification of miRNAs present in samples across a wide range of RNA input levels.

**Electronic supplementary material:**

The online version of this article (10.1186/s13059-018-1488-z) contains supplementary material, which is available to authorized users.

## Background

MicroRNAs (miRNAs) are small non-coding RNAs of approximately 21–23 nucleotides that control the expression of most genes and are involved in many biological processes. Dysregulation of miRNA expression or biogenesis has been implicated in cancer and other diseases [1]. The ability to accurately measure absolute amounts of all miRNAs is important for understanding their biological functions as well as for discovering miRNA biomarkers, developing miRNA-mimic drugs, and identifying miRNA therapeutic targets.

Next-generation sequencing (NGS) is the only approach that allows both discovery of novel miRNA sequences—including sequence variants such as isomiRs—and expression profiling of miRNAs. It is widely believed that NGS will increasingly displace other expression profiling methods in both research and clinical diagnostic applications [2]. Besides its high cost, the major problem preventing the adoption of NGS for routine expression profiling of miRNAs is its poor accuracy (bias) in quantifying many miRNA sequences: most individual miRNAs are underrepresented as a proportion of total sequence reads, in some cases by as much as 10^4^-fold relative to their true abundance within a sample [3–7]. In cases where sequencing bias leads to some miRNAs being under-detected or undetectable in particular samples, NGS can provide misleading or useless results [5, 7, 8]. Indeed, it is likely that many important miRNAs remain to be discovered because they are not incorporated by current library preparation methods [9].

**Fig. 1:**
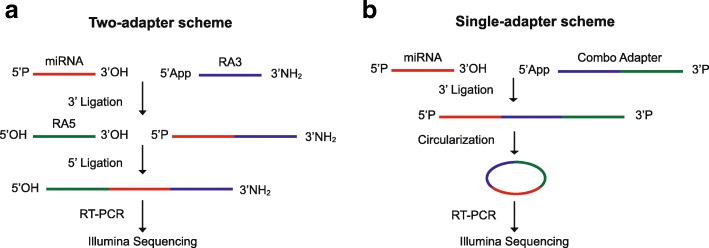
Ligation schemes for sequencing library preparation. **a** Two-adapter ligation; **b** single-adapter ligation plus circularization (RealSeq®-AC)

There is compelling evidence that most of this sequencing bias results from inefficient and sequence-dependent ligation of miRNAs with the two sequencing adapters, a key step in the most widely used methods of sequencing library preparation (Fig. 1a). Several approaches to addressing this adapter ligation bias have been developed [4–6, 8, 10–13], including partially randomizing sequences at either the ends [14] or internal regions of adapters [8]. The latter approach allows co-folding of the adapters with the miRNA and ligation products, increasing ligation efficiency by bringing the ends of the adapters and miRNA closer together [8]. As demonstrated below, however, methods incorporating these approaches remain significantly biased.

## Results

Here we describe a new, more effective approach to reducing ligation bias. In our approach (Fig. 1b), we incorporate the sequences of both standard sequencing adapters into a single adapter. This single adapter is ligated to the 3′ end of the miRNA and the miRNA–adapter ligation product is then circularized via intramolecular ligation. Before circularization of the miRNA–adapter ligation product, a blocking oligonucleotide is ligated to the 5′-preadenylated end of the remaining unligated adapter to inhibit adapter circularization, which would be a template for adapter-dimer amplification. RT-PCR amplification of the resulting circles generates a library suitable for use in standard next-generation platforms. With this approach, the inefficient, intermolecular 5′-adapter ligation step is replaced by a highly efficient intramolecular ligation.

To assess the value of this approach, we first identified eight miRNAs for which detection with a two-adapter ligation approach is accompanied by a wide range (~ 2^16^-fold) of bias levels (Additional file 1: Figure S1). Using this set of eight miRNAs we developed a highly efficient ligation and circularization protocol that minimized differences in ligation and circularization efficiency among these eight miRNAs, with < 20% variation in single-adapter ligation efficiency and < 25% variation in circularization efficiency (Additional file 1: Figure S2).

**Fig. 2:**
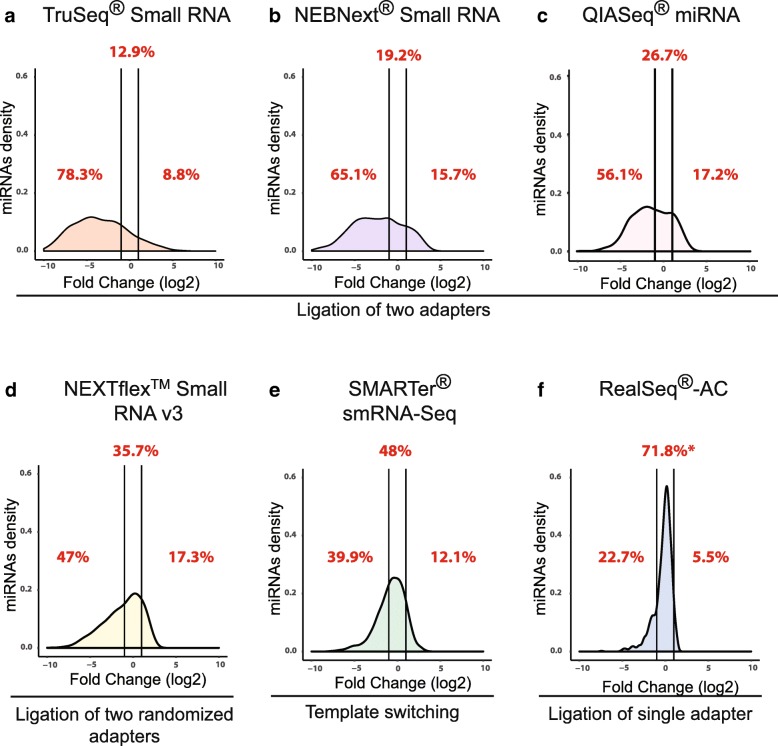
Bias in miRNA detection using various small-RNA library preparation kits. For each kit, sequencing libraries were prepared from the miRXplore™ pool and sequenced; the sequence data were then used to calculate fold-deviations from the equimolar input and plotted as log_2_ values. Densities of miRNAs within a two-fold deviation from the expected values (between *vertical lines*) are considered unbiased according to [8]. Under-represented, over-represented, and accurately quantified percentages of miRNAs are shown in *red font*. Results for two-adapter schemes are **a** TruSeq® Small RNA, **b** NEBNext®, and **c** QIAseq. **d** NEXTFlex™, a scheme using two adapters with randomized sequences. **e** SMARTer, which uses template switching. **f** RealSeq®-AC, which uses a single-adapter and circularization (*p value vs other kits < 0.005)

Based on this novel single-adapter approach, we have developed a new protocol for preparing small-RNA sequencing libraries called RealSeq®-AC (Fig. 1b**)**. To test this protocol and compare its bias in detection of miRNAs to other commercially available library preparation kits, we prepared sequencing libraries in triplicate using RealSeq-AC along with five commercially available library preparation kits: NEBNext® (NEB), TruSeq® (Illumina), NEXTFlex™ (Bioo Scientific/PerkinElmer), QIAseq (Qiagen), and SMARTer (Takara Bio). Libraries were prepared for a pool of 963 synthetic miRNAs, including the eight miRNAs described above, present in equimolar concentrations (miRXplore™ Universal Reference, Miltenyi Biotech). This pool has become established as a standard validation tool for microarray, RT-qPCR, and NGS assays [8]. To determine bias in miRNA quantification, we calculated the fold-deviation from the equimolar input by using the ratio of observed to expected numbers of sequencing reads for each miRNA, and the expected reads are equal for all miRNAs in the pool, as previously described [8]. By this standard measure of bias in miRNA detection, we found that RealSeq®-AC showed significantly less bias (*p* value < 0.005) than the other kits (Fig. 2**,** Additional file 1: Figure S3, Additional file 2: Table S1). Kits employing variants of the two-adapter scheme showed the greatest bias, with most miRNAs being underrepresented (negative log_2_(fold-change)), a much smaller number overrepresented (positive log_2_(fold change)), and between 12.9% and 35.7% accurately quantified (defined as log_2_(fold change) between − 1 and 1, or observed reads within ± 2-fold of expected) (Fig. 2a–d). SMARTer, a ligation-free technique that uses poly(A)-tailing and reverse transcriptase (RT) template switching to detect small RNAs, accurately quantified 48% of miRNAs (Fig. 2e), while RealSeq®-AC accurately quantified 71.8% (Fig. 2f).

**Fig. 3:**
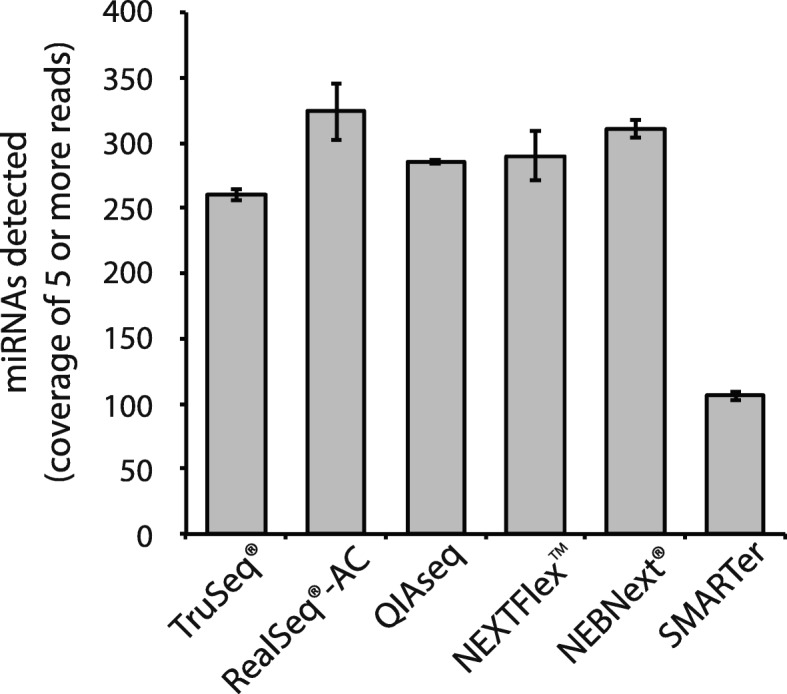
Brain miRNAs detected by different library preparation kits. Sequencing libraries were prepared in triplicate and subsampled to the same number of trimmed reads (200,000 per library), and the average number of high-confidence miRNAs [15] identified is shown. *Error bars* represent the standard deviation of triplicate experiments

The use of a reference pool of synthetic miRNAs provides a straightforward way to measure ligation bias. However, for biological RNA samples, other RNA molecules interfering with the enzymatic reactions can potentially influence the accuracy and sensitivity of miRNA detection. To test for differences in detection of endogenous miRNAs among different library preparation kits, we prepared sequencing libraries in triplicate from a Human Brain Total RNA sample (ThermoFisher) using each of the six kits indicated in Fig. 3. Each library was sequenced and the reads aligned against all miRNAs in miRBase 21 [15] (see also “Methods” and Additional file 2: Table S2). After sub-sampling sequencing reads to analyze the same number of reads for each kit for each member of the triplicate, we found that RealSeq®-AC allowed the detection of the largest number of high-confidence miRNAs, with an average of 324 miRNAs detected with coverage of at least five reads (Fig. 3).

**Fig. 4:**
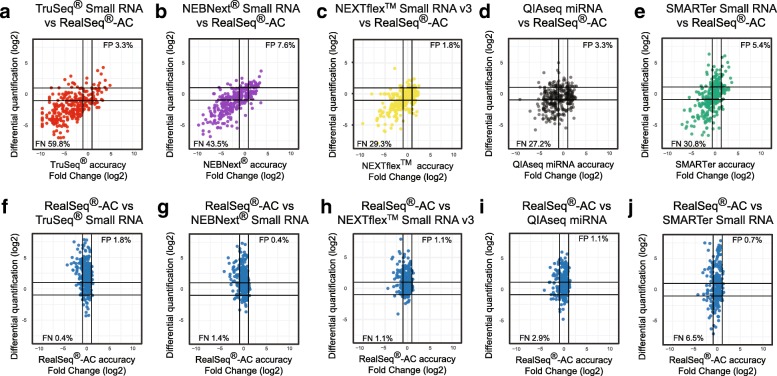
Differential quantification of brain samples between different small RNA library preparation kits. Data obtained with either **a** TruSeq®, **b** NEBNext®, **c** NEXTFlex™, **d** QIAseq, or **e** SMARTer kits were compared with data obtained with RealSeq®-AC to obtain differential quantification (log_2_) values for 276 high-confidence miRNAs. These values were plotted against the accuracy of detection of that miRNA when profiling the equimolar pool of synthetic miRNAs (Fig. 2a–c). **f–j** The reverse comparison, with the differential quantification of RealSeq®-AC versus each of the other kits plotted against the accuracy of RealSeq®-AC when quantifying the synthetic pool of miRNAs. *FN* false negative, *FP* false positive. See Methods for more details

To compare miRNA profiles of biological samples with “profiles” obtained from the synthetic pool, we selected, from a set of 542 human miRNAs that have been designated as high-confidence miRNAs based on experimental evidence [15], all miRNAs that are also represented in the miRXplore pool (276 miRNAs). In profiling the expression of these 276 miRNAs within the brain sample, we found significant differences in quantification among the various kits (Fig. 4). For example, comparing RealSeq®-AC and SMARTer, 186 (67.4%) of the 276 high-confidence miRNAs present in the synthetic pool showed more than a twofold difference in quantification between the two kits (Fig. 4e). Of these 186 differentially quantified miRNAs, 100 (53.8%) showed a strong detection bias (more than twofold) when the synthetic pool was profiled with the SMARTer kit, while only 10.7% had such bias with RealSeq®-AC (Fig. 4e, f). Comparing the differences in miRNA quantification between RealSeq®-AC and the other small RNA library preparation kits and assessing these differences according to each kit’s accuracy of detection of the synthetic pool, we calculated the percentage of miRNAs that each kit either fails to detect (false negatives) or for which it overestimates the abundance (false positives) due to incorporation bias. RealSeq®-AC showed the lowest rate of false positive and false negative measurements (Fig. 4). This suggests that most of the differences seen among library preparation kits when profiling biological samples can be attributed to bias in detection [16].

The development of small RNA library preparation kits has been focused mainly on the detection of miRNAs. This narrow focus has hindered the detection of other classes of small RNAs. Different classes of small RNAs contain features that could further increase their bias in detection by current techniques (e.g., piRNAs have 2′-OMe modifications at their 3′ ends). To further characterize the ability of RealSeq®-AC to efficiently detect other classes of small RNAs, we profiled an RNA reference sample (Universal miRNA Reference kit, Agilent) comprised of RNAs from nine human tissues and cell lines, including adult testis and a testis cell line. It has been demonstrated that piRNAs play important roles during spermatogenesis, where their levels of expression are increased [17]. This sample thus contains a high percentage of piRNAs with 2′-OMe modifications at their 3′ ends. We found that, of the reads that map to small RNAs, the percentage of piRNA reads is higher for RealSeq-AC (17.9%) than for the three other kits tested (QIAseq 7.1%, NEXTFlex™ 5.5%, and TruSeq® 6.2%) (Additional file 1: Figure S4). The apparently larger bias of other kits against non-miRNA small RNAs compared to RealSeq®-AC may contribute to the fact that a larger percentage of their small-RNA reads map to miRNAs.

Several recent studies comparing NGS and RT-qPCR for miRNA quantification have found poor agreement between these platforms [18–22]. To perform a similar comparison with RealSeq®-AC and RT-qPCR, we identified the 15 most-abundant brain miRNAs according to RealSeq®-AC (Additional file 2: Table S3). From these, we selected four miRNAs that show very disparate abundances when quantified by the various sequencing kits and quantified them by RT-qPCR (Additional file 1: Figure S5 and Additional file 2: Table S4). The rank order of relative abundance of these four miRNAs as determined by RealSeq®-AC was the same as that determined by RT-qPCR, whereas all other kits tested ranked the selected miRNAs differently to RT-qPCR. The kit with the best correlation between quantification by sequencing and by RT-qPCR was RealSeq®-AC (*r* = 0.89). While RT-qPCR procedures can themselves be biased, these results suggest that the previously reported poor correlation between NGS and RT-qPCR data may have been largely due to bias introduced by the procedures used for sequencing library preparation.

Based on the above results showing its better accuracy in quantification of miRNAs by using a single-adapter and circularization, we tested whether RealSeq®-AC could detect and profile a larger number of different miRNAs in an RNA sample than other small RNA sequencing library preparation kits. Using RealSeq®-AC and TruSeq®, we prepared sequencing libraries from a reference sample of total RNA (Agilent) obtained from nine different human tissues and cell lines. We sequenced both libraries to a coverage of ten million reads and counted the number of miRNAs identified (with ten or more reads of each) by each kit at different sequencing coverages by random subsampling (Additional file 1: Figure S6). At the deepest sequencing coverage of ten million reads, we identified 641 miRNAs using RealSeq®-AC and only 588 miRNAs using TruSeq®. Thus, by using RealSeq®-AC we detected ~ 10% more miRNAs than TruSeq® at the same coverage. Such an increase in coverage allows the reliable quantification of a larger number of miRNAs for differential expression studies and highlights the importance of accuracy in detection even when performing relative profiling measurements.

**Fig. 5:**
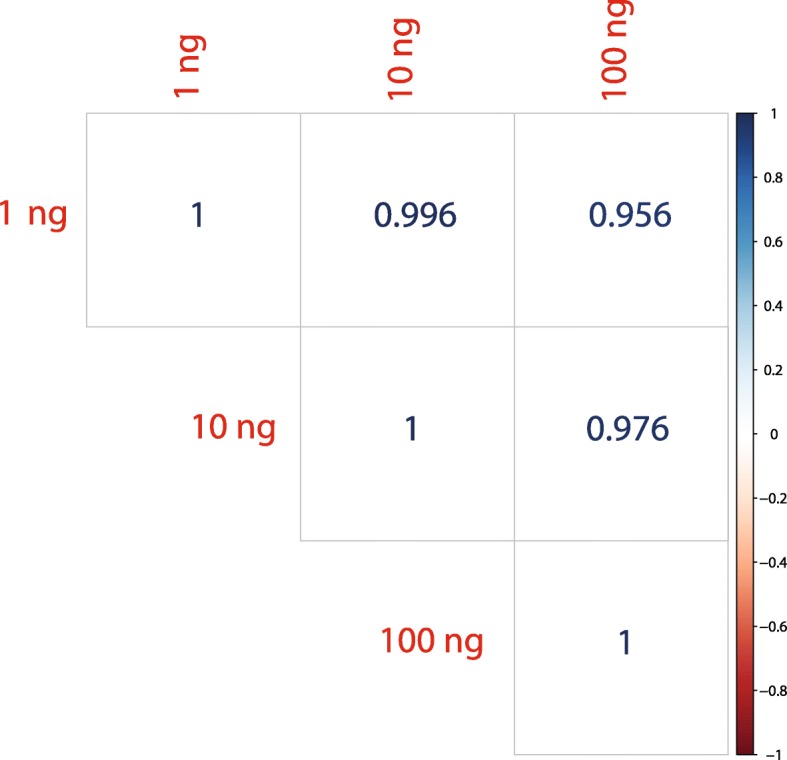
Correlation of miRNA reads between libraries created with 100 ng, 10 ng, or 1 ng inputs of Human Reference RNA (Agilent). Raw reads mapping to miRNAs were used to calculate the Pearson correlation between libraries

The recent surge of interest in detecting extracellular RNAs has increased the need for accurate measurement of miRNA representations from samples with extremely low concentrations of miRNAs. Due to the inefficient and biased to particular miRNA sequences ligation steps, current library preparation methods that use two-adapter ligation techniques require a high number of PCR amplification cycles to reliably detect miRNAs from low-input samples. While PCR bias is much less of a factor than adapter ligation bias in contributing to inaccuracy of detecting miRNAs, the known bias of PCR toward favoring GC-rich, more abundant, and highly similar miRNA sequences can further hinder the accurate quantification of miRNAs [23]. Also, for low input samples the high number of PCR cycles required can drastically increase the risk of cross-contamination of sequencing samples. For example, several reports have questioned the identification of circulating miRNAs of dietary origin, with some publications suggesting that cross-contamination with other samples is to blame for their identification [24]. Considering the high efficiency of the ligation steps by detecting miRNAs with our approach using a single-adapter and circularization, we aimed to measure the robustness of detection of miRNAs across different input levels and cycles of PCR. For this experiment we profiled a human reference RNA with inputs from 100 to 1 ng in tenfold dilution steps. Figure 5 shows the strong correlation in measuring miRNA levels between different sample dilutions while limiting the number of PCR cycles to less than 20. All sequencing libraries from 100 to 1 ng were prepared with a gel-free protocol that includes only a single bead-based purification step.

## Discussion

The level of bias in NGS quantification of miRNAs in currently used library preparation remains very high. This bias hampers the detection of miRNA that could be reliable biomarkers and lowers the reproducibility of sequencing results. It has been shown that the main contributors to library preparation bias are the ligation steps [4, 5], yet almost all commercially available library preparation kits use the same approach of ligating two adapters to the miRNAs, with similar protocols and reagents.

Here we introduce an alternative approach that provides the best accuracy in detection of miRNAs among all tested technologies (Fig. 2). Accurate detection of miRNAs not only provides more accurate relative and absolute quantification, it also enables the discovery of novel miRNAs and other small RNAs. The improved accuracy of RealSeq®-AC is due to two features: i) the use of a single adapter containing the sequences of both 5′ and 3′ standard sequencing adapters in the 3′ ligation step; and ii) replacement of the intermolecular ligation of the 5′ adapter in the two-adapter ligation methods, with a highly efficient and unbiased circularization step.

Due to a high rate of adapter dimer formation, several small RNA library preparation protocols require gel-purification steps to remove adapter dimers. The use of gel purification diminishes the reproducibility of small RNA sequencing experiments: any variability in the efficiency of separation will result in differential recovery of specific small RNA between samples. RealSeq®-AC provides very little formation of empty adapter/adapter dimer, allowing gel-free purification of libraries with as low as 1 ng of input total RNA (Fig. 4). This gel-free purification allows for higher reproducibility of sequencing results between libraries.

RealSeq®-AC differs from previously described single-adapter and circularization methods [25–31] in several respects. (1) The previous single adapter methods use ligation of a standard 3′ sequencing adapter followed by reverse-transcription of the miRNA–adapter ligation products with an RT primer that contains sequences of both adapters used in the two-adapter ligation methods. The resulting cDNA is then circularized and the circular cDNA is PCR-amplified using a pair of PCR primers specific for the standard adapter sequences. In contrast to these methods, RealSeq®-AC incorporates both standard primers into the single adapter rather than the RT primer. (2) In RealSeq®-AC, the combo adapter has RNA nucleotides at its 3′ end that provide circularization by ligating the RNA ends of miRNA–adapter, whereas in the other methods it is the cDNA that is circularized. The efficiency of intramolecular ligation (circularization) is significantly higher between RNA ends than between DNA ends [31, 32]. (3) RealSeq®-AC directly incorporates the miRNA sequences into the sequencing libraries whereas other single-adapter methods incorporate the RT products of these sequences. Thus, any premature stopping of reverse transcription will contaminate the sequencing libraries with truncated copies of RNA sequences. Furthermore, reverse transcriptase can add non-templated nucleotides at the 3′ terminus of the cDNA [25, 30] that may be mistakenly identified as isomiR variants of the miRNAs.

## Conclusions

Due to bias in ligation of adapters during sequencing library preparation, current NGS methods underestimate the abundance of most known miRNAs. To address this issue, we have developed a novel single-adapter ligation and circularization-based method for preparation of small RNA sequencing libraries, RealSeq®-AC, that is user-friendly and requires no gel-electrophoresis steps. By greatly reducing incorporation bias, RealSeq®-AC allows detection of a larger variety of miRNAs and other small RNAs in biological samples with more accurate quantification than other available sequencing methods. Its high accuracy in detection also allows a robust quantification with high correlation across several logs of dilution.

## Methods

### Oligonucleotides

**Table:** 

Single-adapter	AppTGGAATTCTCGGGTGCCAAGG/idSp/idSp/rGrUrUrCrArGrArGrUrUrCrUrArCrArGrUrC
Blocking oligo	/5BiotinTEG/TTCGACGATC
Blocking splint	/5AmMC6/GAGAATTCCATTTTTTTTTT/3AmMO/
RT primer	CCTTGGCACCCGAGAATTCCA
Forward PCR primer	AATGATACGGCGACCACCGAGATCTACACGTTCAGAGTTCTACAGTCCGACGATC
Reverse index PCR primer*	CAAGCAGAAGACGGCATACGAGAT**CGTGAT**GTGACTGGAGTTCCTTGGCACCCGAGAATTCCA

*The sequence shown in bold corresponds to one of the 48 standard Illumina-compatible barcodes. idSp is 1′, 2′-dideoxyribose (dSpacer). 5AmMC6 is a 5′ amino modifier with six carbons. 3AmMO is a 3′ amino modifier. 5BiotinTEG is a 5′ biotin with an extender spacer arm. All DNA oligonucleotides were obtained from IDT

### Library preparation protocol (RealSeq®-AC)

#### Single-adapter adenylation

The 5′-phosphorylated single-adapter was pre-adenylated using a 5′ DNA adenylation kit (NEB) according to the manufacturer’s recommendations.

#### Single-adapter ligation to 3′ end of miRNAs

Ligation of the pre-adenylated single-adapter to the 3′ end of the miRNAs was performed with either truncated RNA ligase 2 [Rnl2(tr)], Rnl2(tr)K227Q, or Rnl2(tr)KQ (NEB), with similar results obtained with all three enzymes. The reaction included 1 pmol of the miRXplore pool or 1 μg of human brain total RNA (ThermoFisher, AM7962) or 1 μg Universal miRNA Reference Kit (Agilent, 750,700), 1× T4 RNA ligase buffer, 200 units of Rnl2(tr), 40 units of RNase OUT (Life Technologies/ThermoFisher), 15% PEG 8000, and 75 ng of single-adapter, in a 10 μl reaction volume. The reaction mix was incubated for 1 h at 25 **°**C in a thermocycler followed by 10 min at 65 **°**C.

#### Adapter blocking

To inhibit the amplification of unligated single-adapter, a blocking oligo was ligated to the remaining unligated single-adapters after the ligation of miRNAs was completed. A blocking reaction mix was prepared with 10 μl of the adapter–miRNAs ligation reaction, 2.5 μl of a 10-μM mix of blocking oligo and blocking splint, 400 units of T4 DNA ligase, and 1 unit of T4 polynucleotide kinase (NEB) in 1× T4 RNA ligase buffer in a 20-μl total volume. This reaction mix was incubated for 1 h at 37 **°**C and 20 min at 65 **°**C.

#### Circularization

To circularize the miRNA–adapter products, 10 units of T4 RNA ligase 1 and 450 μM ATP (sodium salt at pH 7.0 from NEB) were added to the 20 μl reaction mixture from the adapter blocking step for a final reaction volume of 22 μl. This reaction mix was incubated at 37 **°**C for 1 h.

#### RT-PCR

Reverse transcription of the circular miRNA–adapter templates was performed with SuperScript IV (Invitrogen). The reaction mix included 22 μl from the circularization reaction, 1× SSIV Buffer (Invitrogen), 40 units of RNase OUT (Life Technologies), 1.25 μM RT primer, 5 mM dNTPs, and 200 units of SuperScript IV in a 40 μl total reaction volume. The reaction mix was incubated for 30 min at 50 **°**C followed by 10 min at 80 **°**C. PCR was performed with LongAmp® Hot Start Taq DNA polymerase (NEB). The reaction included 40 μl from the RT reaction, 1× LongAmp® Taq Reaction Buffer (NEB), 3 mM dNTPs, 0.7 μM forward PCR primer, 0.7 μM reverse index primer, and 10 units of LongAmp® Hot Start Taq DNA polymerase in a 100 μl reaction volume. The PCR reaction was performed for either 5 cycles for the miRXplore pool, or 7, 10, 13, or 16 cycles for 1 μg, 100 ng, 10 ng or 1 ng 3RNA samples respectively. PCR included a first step at 94 **°**C for 30 s, and 5, 7, 10, 13, or 16 cycles of 94 **°**C for 15 s, 62 **°**C for 30 s, and 70 **°**C for 15 s, with a final step at 70 **°**C for 5 min.

#### Library preparation for other kits

Experiments with NEBNext® (NEB), TruSeq® (Illumina), NEXFlex™ (Bioo Scientific), QIAseq (Qiagen), and SMARTer (Takara Bio) were performed following the manufacturers’ recommendations. For all kits, 1 pmol of the miRXplore™ Universal Reference (Miltenyi Biotec) was used as input to test accuracy in detection (Figs. 2 and 3). Brain total RNA (1 μg) was used for Fig. 3 for all kits with the exception of QIAseq, where 500 ng of total RNA was used as per the manufacturer’s recommendations. Libraries were prepared in triplicate for all experiments. To determine concentration and quality of libraries, all libraries were analyzed with an Agilent D1000 ScreenTape on a 2200 TapeStation instrument (Agilent) and then quantified with a Qubit dsDNA BR Assay kit on a Qubit 3.0 instrument.

#### RNA samples

Experiments to test accuracy of detection (bias) were performed with 1 pmol of the miRXplore™ Universal Reference (Miltenyi Biotec), which contains equimolar amounts of 963 RNAs that match mature miRNAs. Experiments profiling total RNA were performed with 1 μg of reference Human Brain Total RNA (Life Technologies/ThermoFisher) or with 1 μg Universal miRNA Reference Kit (Agilent).

#### Sequencing

Triplicate libraries for each input and kit, with the exception of brain total RNA with the NEXTFlex™ kit that was performed in duplicates, were pooled at equimolar concentrations and sequenced in Illumina MiSeq or Illumina NextSeq instruments with single-end reads of 36 nucleotides (MiSeq) or 50 nucleotides (NextSeq) following the manufacturer’s recommendations. Libraries were mixed with 5% PhiX.

#### Data analysis

FastQ files were trimmed of adapter sequences by using Cutadapt (10.14806/ej.17.1.200) with the following parameters: cutadapt -a TGGAATTCTCGGGTGCCAAGG -m 15. Trimmed reads were aligned to the corresponding reference by using Bowtie2 [33]; data for Fig. 2 were obtained by alignment to a reference file containing the sequences of all miRNAs included in the miRXplore pool; data for Figs. 3 and 4 were obtained by aligning trimmed reads to a reference containing the sequences of all the high-confidence miRNAs [15]. Data for Additional file 1: Figure S5 were obtained by aligning reads from the Universal miRNA Reference Kit libraries to a reference database of small RNAs (YM500v3 [34]). Analysis for Fig. 4 included differential quantification between RealSeq®-AC and each of the other kits calculated as the log_2_-fold difference; these values are represented on the y-axis (differential quantification) and plotted against the accuracy of detection for each of the 276 high-confidence human miRNAs for each of the kits tested, as determined by quantification of each miRNA within the miRXplore™ pool (Fig. 2 data). Data are available at the NCBI GEO with accession number GSE107304 [35].

#### RT-qPCR

Data for Additional file 1: Figure S4 and Additional file 2: Table S4 were generated with miR-ID® assays [36] for miRNAs hsa-miR-26a-5p, hsa-miR-125b-5p, hsa-miR-16-5p, and hsa-miR-29a-3p with 10 ng reference Human Brain Total RNA following the manufacturer’s recommendations. Absolute quantification to determine the amounts of each miRNA present in the total RNA sample was performed by comparison of the Cq values obtained from brain total RNA with standard dilution curves of the corresponding synthetic miRNA prepared over an 8-log concentration range (200 pM–20 aM).

## Additional files

Additional file 1: **Figure S1.** Sequencing results of a library prepared using the TruSeq® Small RNA kit (Illumina) with 1 pmol of synthetic miRNAs (miRXplore™ Universal Pool) as input. **Figure S2.** Ligation and circularization efficiency of a single-adapter to the group of miRNAs selected in Figure S1. **Figure S3.** Testing accuracy of quantification by sequencing of eight test miRNAs using TruSeq® and RealSeq®-AC kits. **Figure S4.** Percentage of reads corresponding to different classes of small RNAs when sequencing a human reference sample (Agilent). **Figure S5.** Quantification by RT-qPCR and the various sequencing methods of four of the most abundant brain miRNAs according to RealSeq®-AC. **Figure S6.** Number of miRNAs identified by TruSeq® and RealSeq®-AC in a human total RNA reference sample (Universal miRNA Reference kit, Agilent) that was sequenced to high coverage (ten million reads). (PDF 472 kb)

Additional file 2: **Table S1.** *P* values obtained from a two-sample *t*-test between the percentage of unbiased miRNAs measured with RealSeq-AC (in triplicate experiments) against five other library preparation kits. **Table S2.** Analysis of miRNA sequencing data for a reference human brain sample (Life Technologies/ThermoFisher) using different sequencing library preparation kits. **Table S3.** The most abundant brain miRNAs according to RealSeq®-AC. **Table S4.** RT-qPCR validation of abundant brain miRNAs levels (in pM). (PDF 31 kb)
