# Supplementary material for: Decreasing miRNA sequencing bias using a single adapter and circularization approach
Source: Genome Biol. 2018 Sep 3;19:105. doi: 10.1186/s13059-018-1488-z (PMC6120088; doi:10.1186/s13059-018-1488-z)
Supplement: Supplementary file 1 — Figure S1. Sequencing results of a library prepared using the TruSeq® Small RNA kit (Illumina) with 1 pmol of synthetic miRNAs (miRXplore™ Universal Pool) as input. Figure S2. Ligation and circularization efficiency of a single-adapter to the group of miRNAs selected in Figure S1. Figure S3. Testing accuracy of quantification by sequencing of eight test miRNAs using TruSeq® and RealSeq®-AC kits. Figure S4. Percentage of reads corresponding to different classes of small RNAs when sequencing a human reference sample (Agilent). Figure S5. Quantification by RT-qPCR and the various sequencing methods of four of the most abundant brain miRNAs according to RealSeq®-AC. Figure S6. Number of miRNAs identified by TruSeq® and RealSeq®-AC in a human total RNA reference sample (Universal miRNA Reference kit, Agilent) that was sequenced to high coverage (ten million reads). (PDF 472 kb) [file 13059_2018_1488_MOESM1_ESM.pdf]

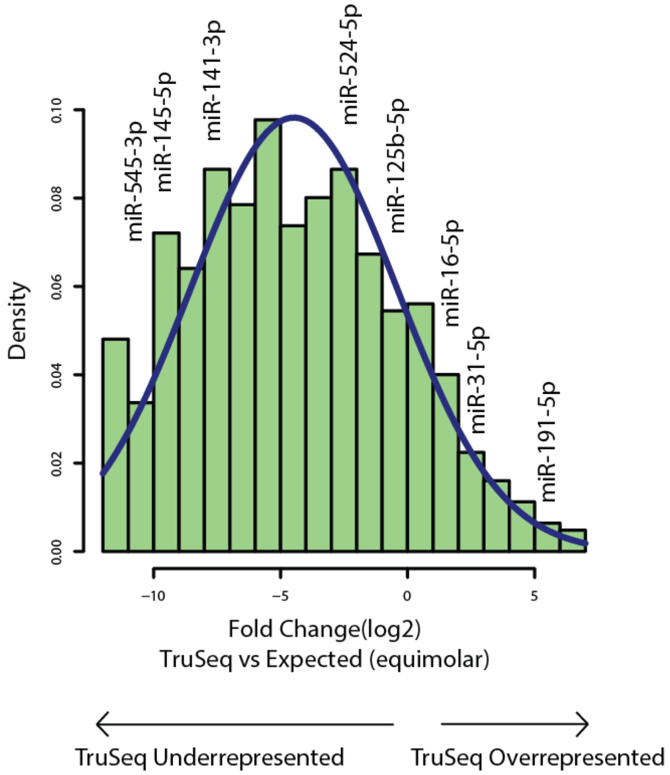

Additional File 1: Figure S1. Sequencing results of a library prepared using the TruSeq® Small RNA kit (Illumina) with 1 pmol of synthetic miRNAs (miRXplore™ Universal Pool) as input. The indicated 8 miRNAs that show a wide range of bias in detection for this method were used to optimize RealSeq-AC for minimal bias.

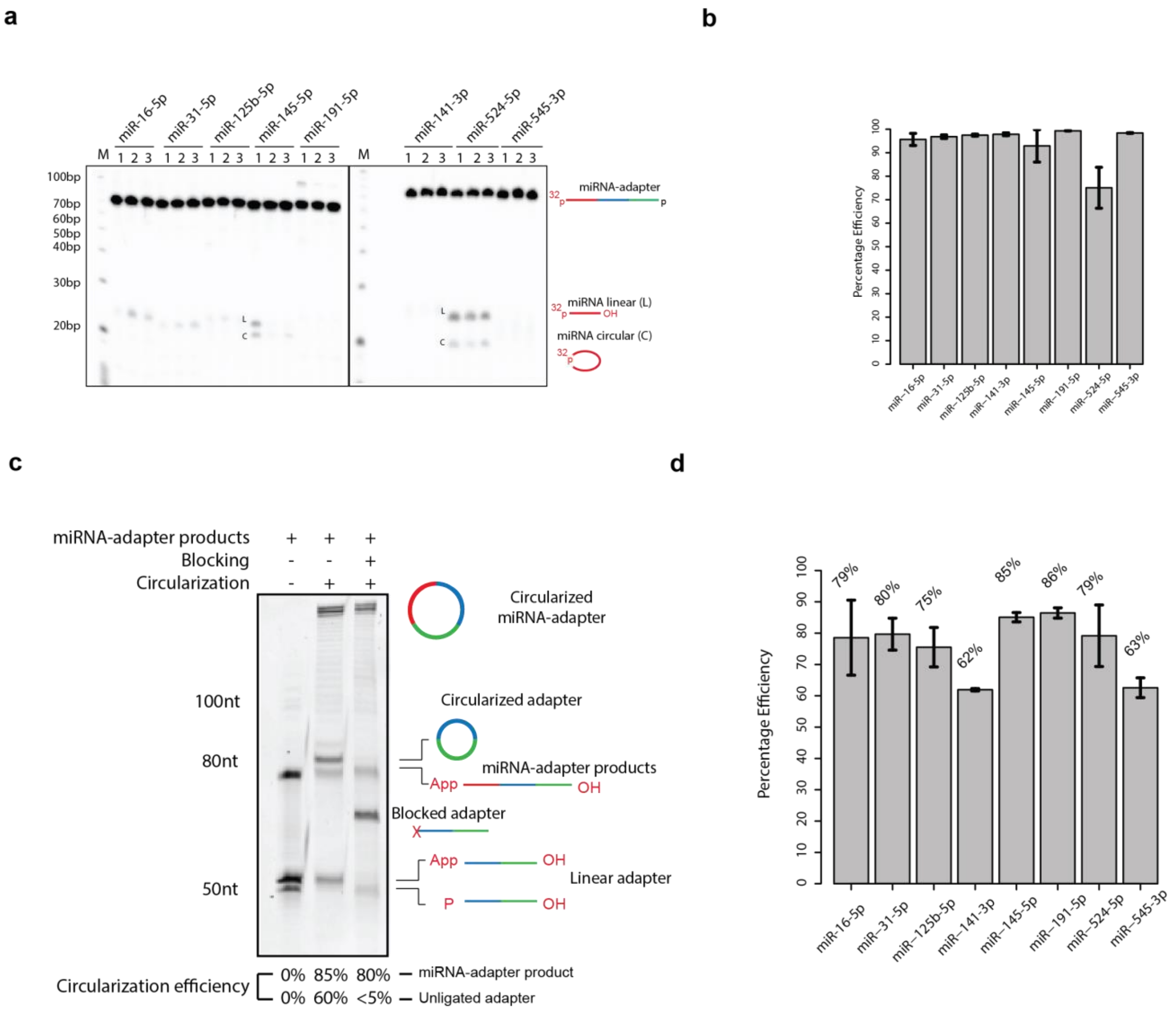

Additional File 1: Figure S2. Ligation and circularization efficiency of a single-adapter to the group of miRNAs selected in Supplementary Fig. 1. (**a**) Efficiency of miRNA ligation to the single-adapter. Ligation reactions (see Online Methods for details) performed in triplicate were resolved using 15% PAGE (8 M Urea), miRNAs were radiolabeled with ^{32}P, and ligation products were quantified using a phosphorimager. (**b**) Quantification of ligation yields from (a). (**c**) Yields of various species when a pool of all 8 selected miRNAs is ligated to the single-adapter (in multiplex), as resolved by 15% PAGE. Lane 1 shows only ligation products, lane 2 includes circularization of ligation products, and lane 3 also includes adapter blocking (see Online Methods for details). (**d**) Circularization efficiency of miRNA-adapter ligation products for the eight individual miRNAs, with the circularization reaction performed here in singleplex for each miRNA-adapter ligation product. Error bars represent standard deviations of technical triplicates.

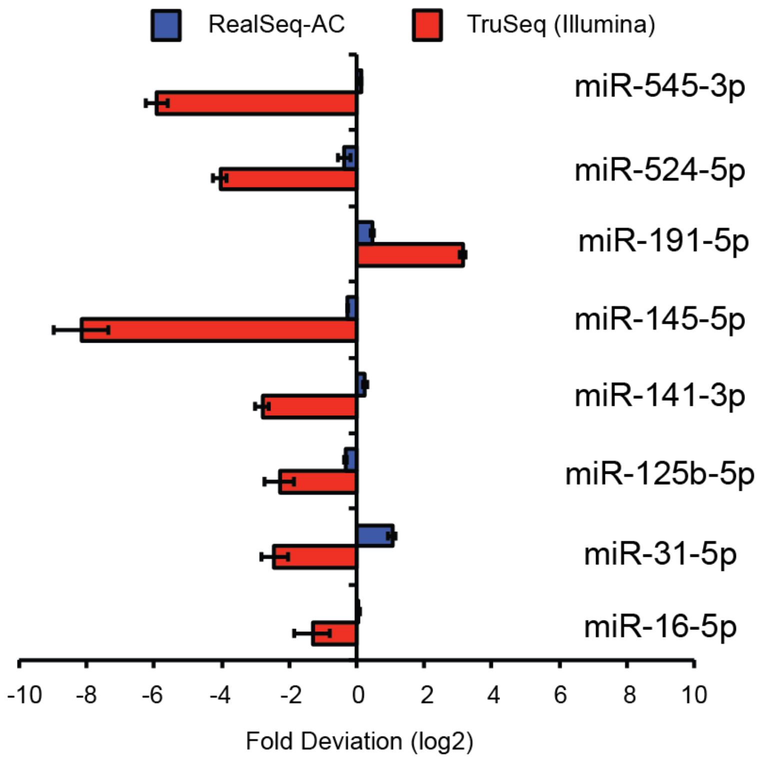

Additional File 1: Figure S3. Testing accuracy of quantification by sequencing of eight test miRNAs using TruSeq® and RealSeq®-AC kits. Sequencing libraries were prepared in triplicate using as input 1 pmole of miRXplore™ Universal Reference equimolar pool of miRNAs. Purified libraries were sequenced on the Illumina MiSeq platform. Trimmed sequencing reads were aligned to a custom miRNA reference (Bowtie2[23]). Reads mapping to the eight selected miRNAs were counted and fold-deviations from the equimolar input were calculated and plotted as log2 values.

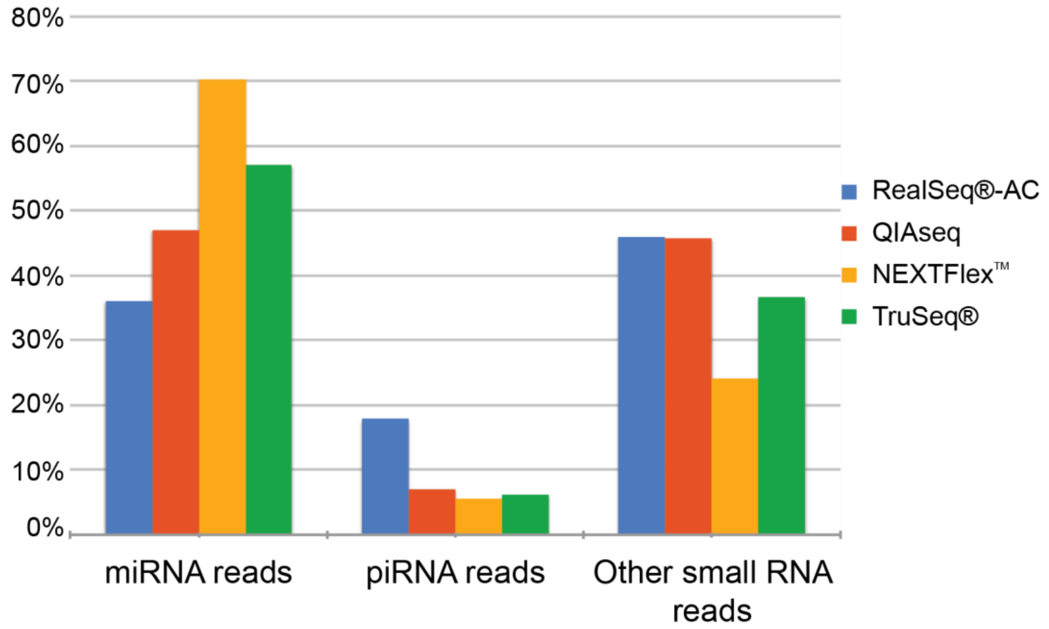

Additional File 1: Figure S4. Percentage of reads corresponding to different classes of small RNAs when sequencing a human reference sample (Agilent). Sequencing results were sub-sampled to 13 million trimmed reads per kit and reads were aligned to the YM500v3 database [36] to identify different classes of small RNAs.

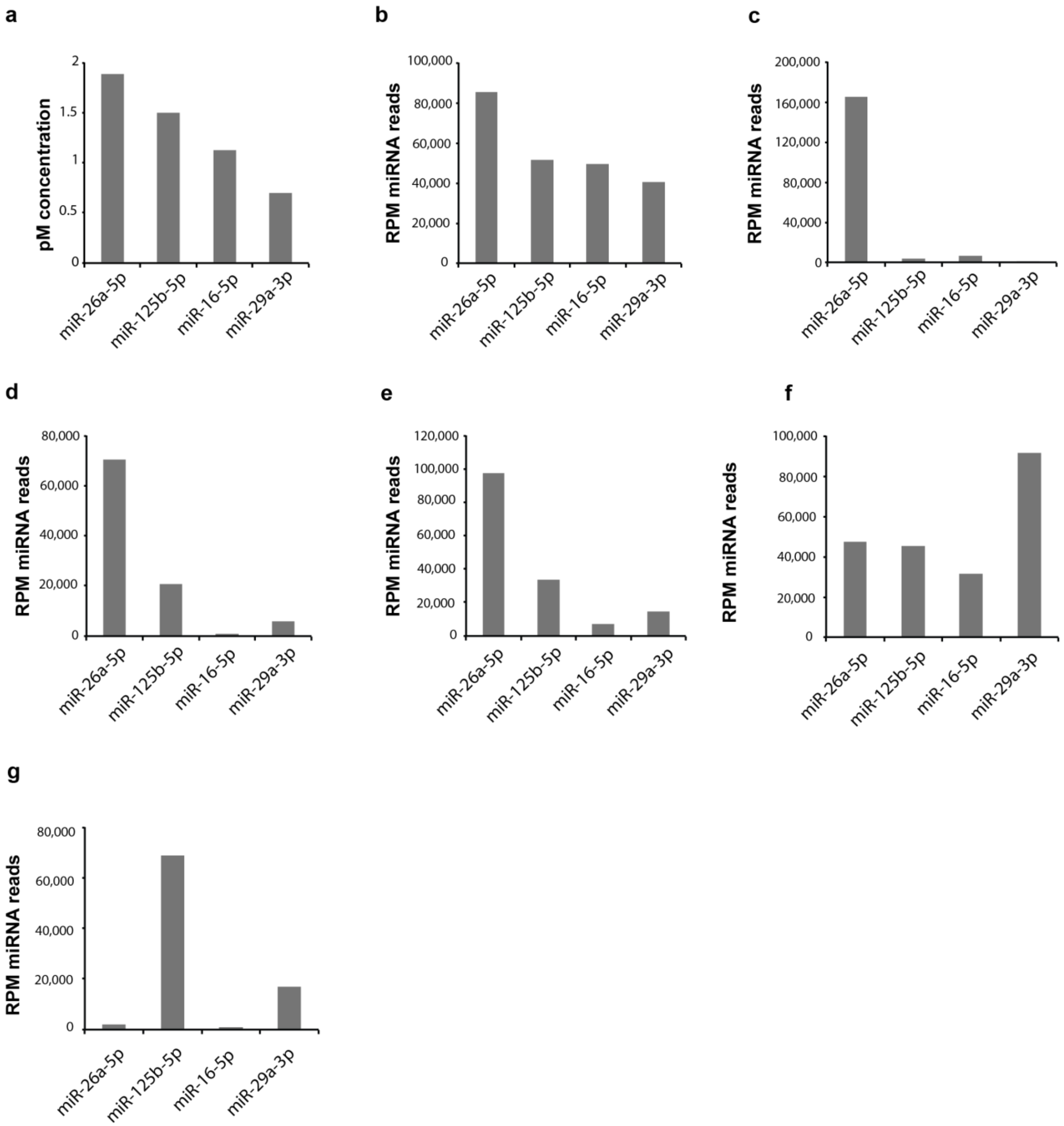

Additional File 1: Figure S5. Quantification by RT-qPCR and the various sequencing methods of four of the most abundant brain miRNAs according to RealSeq®-AC. (**a**) miR-ID® RT-qPCR; (**b**) RealSeq®-AC; (**c**) TruSeq® Small RNA; (**d**) NEBNext® Small RNA; (**e**) NEXTFlex™ Small RNA v.3; (**f**) QIAseq miRNA; (**g**) SMARTer Small RNA. For sequencing experiments, the average number of reads per million (RPM) miRNA reads sequenced was calculated from triplicate experiments.

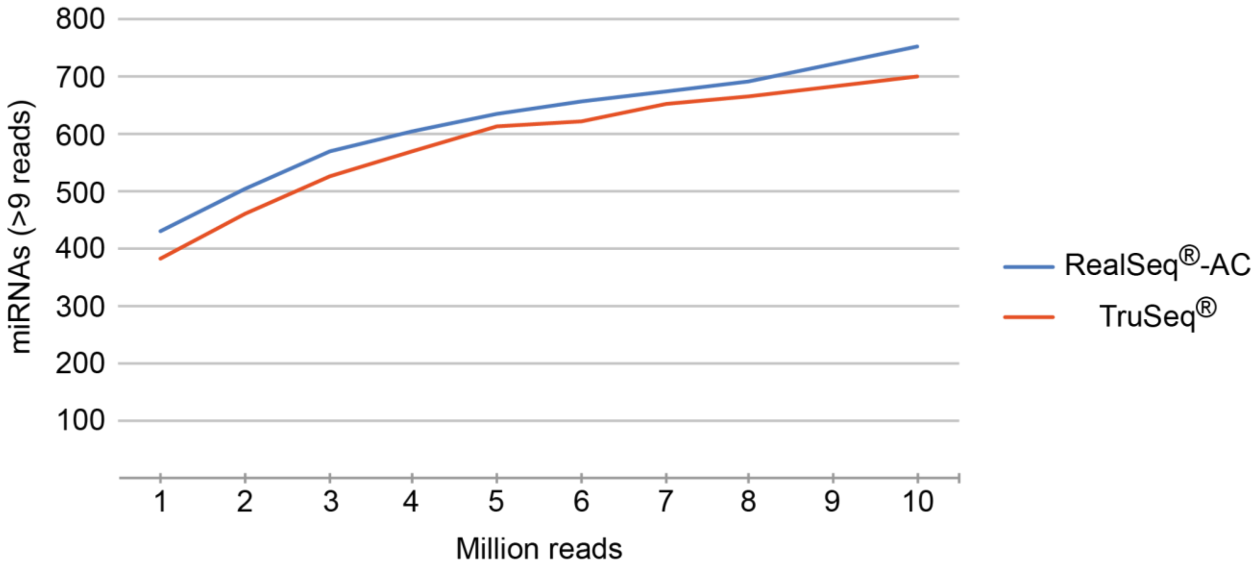

Additional File 1: Figure S6. Number of miRNAs identified by TruSeq® and RealSeq®-AC in a human total RNA reference sample (Universal miRNA Reference kit, Agilent) that was sequenced to high coverage (10 million reads). Total number of different canonical miRNAs identified with 10 or more sequencing reads at different coverage of sequencing (with random subsampling or reads from 1 to 10 million).
