# Supplementary material for: Decreasing miRNA sequencing bias using a single adapter and circularization approach
Source: Genome Biol. 2018 Sep 3;19:105. doi: 10.1186/s13059-018-1488-z (PMC6120088; doi:10.1186/s13059-018-1488-z)
Supplement: Supplementary file 2 — Table S1. P values obtained from a two-sample t-test between the percentage of unbiased miRNAs measured with RealSeq-AC (in triplicate experiments) against five other library preparation kits. Table S2. Analysis of miRNA sequencing data for a reference human brain sample (Life Technologies/ThermoFisher) using different sequencing library preparation kits. Table S3. The most abundant brain miRNAs according to RealSeq®-AC. Table S4. RT-qPCR validation of abundant brain miRNAs levels (in pM). (PDF 31 kb) [file 13059_2018_1488_MOESM2_ESM.pdf]

	**Two sample t-test vs RealSeq®-AC**
**NEBNext®**	0.000009
**NEXTFlex™**	0.000035
**TruSeq®**	0.000002
**SMARTer®**	0.001036
**QIAseq**	0.000007

Additional file 2: Table S1. p-values obtained from a two-sample t-test between the percentage of unbiased miRNAs measured with RealSeq-AC (in triplicate experiments) against five other library preparation kits.

Kit	TruSeq®	NEBNext®	NEXTFlex™	SMARTer®	RealSeq®-AC	QIAseq
**Reads sequenced**	2,127,366	2,220,163	3,645,195	4,626,759	3,121,084	4,600,090
**Reads passing filters**	1,861,982	2,021,889	3,389,169	3,660,906	2,398,556	3,087,960
**Percentage of reads passing filters**	87.5	91.1	93	79.1	76.9	67.1

Additional file 2: Table S2. Analysis of miRNA sequencing data for a reference human brain sample (Life Technologies/ThermoFisher) using different sequencing library preparation kits.

	**RealSeq®-AC top 15 miRNAs**	
	**miRNA**	**RPM miRNA reads**
**1**	**hsa-miR-26a-5p**	85,132
**2**	hsa-miR-9-5p	81,586
**3**	hsa-miR-181a-5p	57,701
**4**	**hsa-miR-125b-5p**	51,667
**5**	**hsa-miR-16-5p**	49,573
**6**	hsa-let-7a-5p	47,046
**7**	**hsa-miR-29a-3p**	40,662
**8**	hsa-let-7f-5p	26,824
**9**	hsa-miR-27b-3p	23,357
**10**	hsa-miR-99a-5p	23,348
**11**	hsa-miR-29b-3p	22,124
**12**	hsa-miR-101-3p	21,072
**13**	hsa-let-7b-5p	18,629
**14**	hsa-miR-143-3p	18,266
**15**	hsa-miR-100-5p	18,153

Additional file 2: Table S3. The most abundant brain miRNAs according to RealSeq®-AC. The average number of reads per million miRNA reads (RPM miRNA reads) was calculated from triplicate experiments. Bold miRNAs are those selected for RT-qPCR validation in Additional file 1: **Fig. S5**.

miRNA	RT-qPCR	RealSeq®-AC	TruSeq®	NEBNext®	NEXTFlex™	QIAseq	SMARTer
hsa-miR-26a-5p	1.89	85,132	164,671	70,549	97,197	47,369	2,119
hsa-miR-125b-5p	1.50	51,667	3,769	20,844	33,486	45,568	68,600
hsa-miR-16-5p	1.13	49,573	6,174	1,130	7,538	31,743	659
hsa-miR-29a-3p	0.70	40,662	1,337	6,128	14,900	91,181	16,910

Additional file 2: Table S4. RT-qPCR validation of abundant brain miRNAs levels (in pM). Four brain miRNAs that are abundant according to RealSeq®-AC were selected for RT-qPCR validation, and absolute levels were determined for each miRNA by RT-qPCR. Numbers shown for NGS kits are Reads per million miRNA reads.
